# Effects of reagent rotation on interferences in the product angular distributions of chemical reactions†
†Electronic supplementary information (ESI) available: Additional figures. See DOI: 10.1039/c5sc03373j



**DOI:** 10.1039/c5sc03373j

**Published:** 2015-10-05

**Authors:** P. G. Jambrina, J. Aldegunde, F. J. Aoiz, M. Sneha, R. N. Zare

**Affiliations:** a Departamento de Química Física I , Facultad de Química , Universidad Complutense de Madrid , 28040 , Spain . Email: aoiz@quim.ucm.es; b Departamento de Química Física , Universidad de Salamanca , Salamanca , Spain; c Department of Chemistry , Stanford University , Stanford , California 94305-5080 , USA . Email: zare@stanford.edu

## Abstract

Differential cross sections (DSCs) of the HD(*v*′, *j*′) product for the reaction of H atoms with supersonically cooled D_2_ molecules in a small number of initial rotational states have been measured at a collision energy of 1.97 eV. These DCSs show an oscillatory pattern that results from interferences caused by different dynamical scattering mechanisms leading to products scattered into the same solid angle. The interferences depend on the initial rotational state *j* of the D_2_(*v* = 0, *j*) reagent and diminish in strength with increasing rotation. We present here a detailed explanation for this behavior and how each dynamical scattering mechanism has a dependence on the helicity Ω, the projection of the initial rotational angular momentum *j* of the D_2_ reagent on the approach direction. Each helicity corresponds to a different internuclear axis distribution, with the consequence that the dependence on Ω reveals the preference of the different quasiclassical mechanisms as a function of approach direction. We believe that these results are general and will appear in any reaction for which several mechanisms are operative.

## Abstract 2

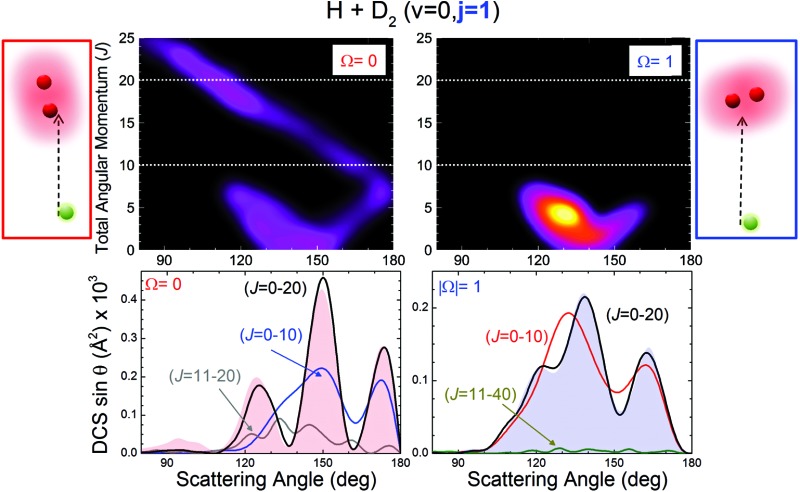
The amplitude of the interference pattern in the angular distributions diminishes with increasing rotational excitation due to the incoherent sum over the helicity reactant states associated with different mechanisms.

## Introduction

The Young's double-slit experiment applied to the interference of single electrons has been considered to be the most beautiful and intriguing experiment in physics^1–3^ and has become a standard example in quantum mechanics (QM) textbooks for illustrating wave–particle duality. In the experiment, electrons, one at a time, are shot against a screen that contains two small openings (slits). The detection of the transmitted electrons results in an interference pattern, similar to that observed when light waves instead of electrons are used. These results give convincing evidence for the probabilistic nature of the quantum process. Moreover, the experiment has also been carried out using heavier particles,^4–9^ such as fullerene molecules, leading to similar conclusions and demonstrating the quantum nature of these large and presumably more classical molecules.

It might be wondered whether such interference effects occur in chemical reactions and, if so, how they affect the reaction observables. In a recent article,^10^ we measured state-to-state angular distributions of the HD product reactively scattered at a collision energy, *E*_coll_, of 1.97 eV, for the benchmark H + D_2_ → D + HD reaction using the photoloc technique.^11,12^ The angular distribution, or differential cross section (DCS), represents the cross section as a function of the scattering angle, *θ*, and its QM value is obtained as the square of a coherent sum of partial waves from the different values of the total angular momentum, *J*. Hence, this summation contains cross terms from different *J* values and could possibly give rise to interferences. However, coherences are usually only important between partial waves characterized by very similar values of *J* and, in many cases, scattering at different angles can be approximately attributed to specific groups of partial waves^13^ similar to that occurring in quasiclassical trajectory (QCT) calculations. This general behaviour encourages simple pictures to be presented for the scattering process in which the nuclear motions are treated classically with no interference effects.

However, the observed angular distributions at *E*_coll_ = 1.97 eV, obtained for low rotational and vibrational excitation of the HD products were dominated by an oscillatory pattern, resembling that from interferences in the double-slit experiment.^10^ These results could not be accounted for by the QCT method, wherein the nuclei are treated as classical particles moving on a potential energy surface (PES) originating from quantum electronic motions, even though it usually constitutes a very good approximation for the dynamical description of the H + D_2_ and many other chemical reactions.^14^ The fact that the measurements could only be reproduced by exact QM calculations (where both nuclei and electrons are treated quantum mechanically) clearly indicated that the observed oscillatory pattern in the angular distribution was caused by a quantum phenomenon. Oscillations in the energy dependent DCS at given fixed angles had been previously measured and attributed to either partial wave resonances^15,16^ or to interferences through a network of quantum bottleneck pathways.^17^ However in this case there are no kinds of resonance and the positions of the peaks do not depend much on small changes on the total energy.

A careful analysis of the reactant's ground rotational state (*j* = 0) allowed us to unequivocally attribute these oscillations to quantum interferences between different underlying classical mechanisms, characterized by well-defined ranges of the total angular momentum and giving rise to scattering at certain angles. By analogy, the PES acts as an interferometer in which the different mechanisms play the role of the pathways in the classical double-slit experiment; interferences are observed whenever two distinct mechanisms lead to products scattered into the same scattering angle. To understand the nature of these phenomena, we carried out a computational analysis in which we shut down the various scattering mechanisms, one at a time, so as to observe the disappearance of the different peaks in the interference pattern. This procedure is analogous to that of successively blocking the various slits in a multiple-slit experiment.

In the present study we demonstrate that the aforementioned oscillatory pattern observed in the DCS is very sensitive to the rotational state of the D_2_ reagent. In particular, we will show that interference patterns like those observed for *j* = 0 collisions are also found in encounters where the reactant molecule is rotationally excited, although the amplitude of the interferences diminishes rapidly with increasing rotational angular momentum of the D_2_ reactant. In the first instance, this diminishing is a consequence of the larger number of |*j*, Ω initial states that add incoherently as the reaction occurs, where the helicity Ω is the projection of *j* on the reactants' approach direction. Although an interference pattern exists for each (*j*, Ω) combination, the different Ω values contribute incoherently and the interference patterns become blurred. For *j* = 0, there is only one Ω value (Ω = 0) and the interference pattern is sharp. As *j* increases, the incoherent sum over different Ω values causes the interference pattern to smooth and diminish in contrast.

We have concentrated on the H + D_2_ reaction for which we have experimental measurements of the DCSs and strong confidence in the accuracy of the PES. However, we believe that our results apply to all chemical reactions in which more than one classical scattering mechanism contributes to the observed product angular distributions.

The article is structured as follows. The Methods section briefly describes the experimental and theoretical methodologies employed in this work. The Results and discussion section presents the main results and the analysis of the theoretical data. Finally, the Conclusion section summarizes the main findings and future scope of the present work.

## Methods

### Experiment

We employ a three-dimensional (3D) ion imaging setup and the photoloc technique, the details of which are described in previous publications.^11,12^ Briefly, a mixture of 3–5% HBr in D_2_ was co-expanded supersonically into a vacuum chamber through a 10 Hz pulsed valve (General Valve, Series 9), with a typical backing pressure of ∼1300 Torr. This leads to the internal and translational cooling of reactants with D_2_ being prepared almost exclusively in the (*v* = 0, *j* = 0, 1 and 2) internal states with a relative population of 0.39 : 0.31 : 0.29, respectively. The reaction is initiated by photodissociating HBr with a focused, 199 nm UV beam (60 cm lens), which generates translationally hot H atoms with two different collision energies; *E*_coll_ = 1.97 eV and 1.51 eV, referred to as fast channel and slow channel, respectively. These two channels arise from the spin–orbit splitting in bromine, and the ratio of the fast channel to the slow channel is 0.84 : 0.16 at the photolysis wavelength of 199 nm. These H atoms react with D_2_ to form HD(*v*′, *j*′) products. After a delay of 15–20 ns, a counter propagating focused UV beam (60 cm lens) is used to probe the HD products state-selectively using a [2 + 1] resonance enhanced multiphoton ionization (REMPI) scheme on the Q branch of the E,F^1^Σ_g_^+^–X^1^Σ_g_^+^ transition. The lab frame speed of the HD ions is measured using a Wiley–McLaren time-of-flight instrument equipped with a position-sensitive detector and converted into a DCS based on the photoloc technique described elsewhere in detail.^12,18^ At the photolysis wavelength used here, *i.e.*, 199 nm, the contribution from the slow channel is very small compared to the fast channel and can therefore be ignored^18^ when converting the speed distribution to DCS.

### Theory

Time-independent quantum mechanics (TIQM) calculations were carried out on the BKMP2 (ref. 19) surface using the coupled-channel hyperspherical method implemented in the ABC code.^20^ To simulate the experimental conditions, the S matrix was obtained for 90 different collision energies in the 1.85–2.12 eV range for *j* = 0, and similarly for *j* = 1 and 2 (*E*_tot_ = *E*_coll_ + *E*_*v*,*j*_ = 2.04 – 2.31 eV) to fully cover the experimental Gaussian distribution centred at *E*_coll_ = 1.97 eV, with 0.1 eV FWHM, and the averaging over the initial rotational states populated in the D_2_ molecular beam. The propagation was performed in 250 sectors from 0.8 to 24.0 *a*_0_, including in the basis all the diatomic energy levels up to 3.15 eV and helicity quantum numbers Ω_max_ = 18. All the partial waves up to *J* = 40 were included. The present TIQM calculations do not include the geometric phase effect, which takes into account the change of sign of the wave function when encircling a conical intersection. For the H + H_2_ reaction, this effect is only expected to show up in the DCSs at energies above *E*_coll_ = 3.5 eV.^21^ Even at 3.26 eV, well above the conical intersection, the comparison between QM calculations and photoloc experiments for H + D_2_ renders an excellent agreement,^22^ providing credibility to calculations without the geometric phase.

Regarding the QCT calculations, three batches of 15 million trajectories were run at *E*_coll_ = 1.97 eV for the H + D_2_(*v* = 0, *j* = 0–2) collisions following the procedures described in a previous publication.^23^ An integration step of 5 × 10^–17^ s and a maximum impact parameter *b* = 1.4 Å were used in the integration of the trajectories. The rovibrational energies of the HD product molecules were calculated by semiclassical quantization of the action and their values were fitted to Dunham expansions in (*v*′ + ½) and *j*′(*j*′ + 1). The (real) *j*′ value was assigned by equating the square of the classical HD rotational angular momentum to *j*′(*j*′ + 1)*ħ* ^2^. Comparison of the internal energy to that given by the rovibrational Dunham expansion for a specific *j*′ value yields the value of *v*′.

Two additional batches of 10 million trajectories each were run at *E*_coll_ = 1.97 eV for the H + D_2_(*v* = 0, *j* = 1 and 2) using the *J*–Ω scheme,^24^ wherein *J* and Ω were sampled in discrete, integer values. In this scheme, *J* is sampled uniformly and, once it has been done, Ω is chosen also uniformly within the range of allowed values –min(*J*, *j*) ≤ Ω ≤ min(*J*, *j*).

### DCS expansion

The QM state-to-state DCS, *I*(*θ*)sin *θ*, was obtained using the following equation1
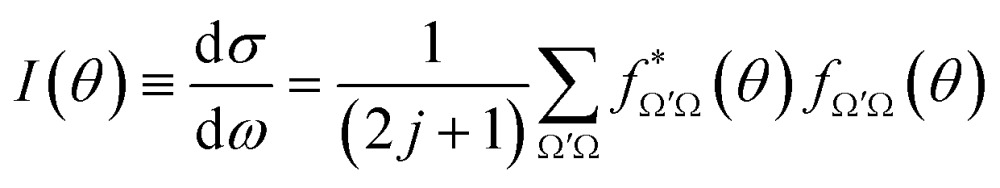
where *f*_Ω′Ω_ is the scattering amplitude defined as:2
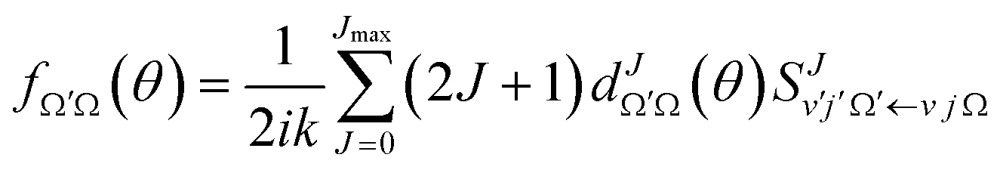
and 
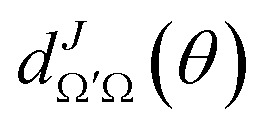
 and 
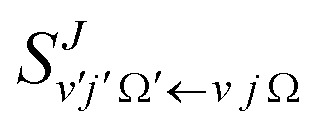
 are the elements of the reduced rotation and scattering matrices respectively, where *J*_max_ is the value of *J* necessary for the convergence of the quantum mechanical calculations. It should be pointed out that the summation over *J* is coherent while the summation over Ω and Ω′ is incoherent. That means that in the DCS we find crossed products between elements of the scattering matrix corresponding to different values of *J* but not between elements corresponding to different values of the helicity. Therefore, without any loss of generality, it is possible to express the DCS as3
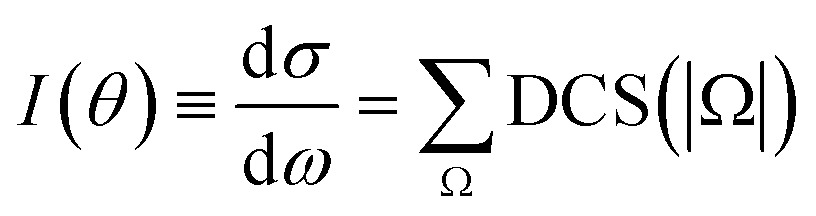
where the different DCS(|Ω|) contributions are calculated as follows:4
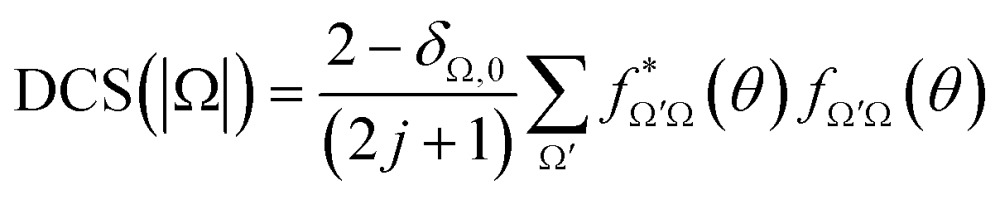
where we have used the fact that DCS(Ω) = DCS(–Ω).

It should be emphasized that, due to the coherences in the summation over *J*, it is not possible to build the analogous DCS(*J*) without including crossed terms between different partial waves. Nevertheless, the summation of *J* in eqn (2) could be restricted to lie within a [*J*_1_, *J*_2_] interval. These DCS will be denoted as DCS(*J*_1_ – *J*_2_) and will be very useful to characterize the importance of coherences between groups of *J*. However, it should be kept in mind that,

5 DCS(*J*_1_ – *J*_3_) ≠ DCS(*J*_1_ – *J*_2_) + DCS[(*J*_2_ + 1) – *J*_3_]

where *J*_1_ < *J*_2_ < *J*_3_.

## Results and discussion

**Fig. 1:**
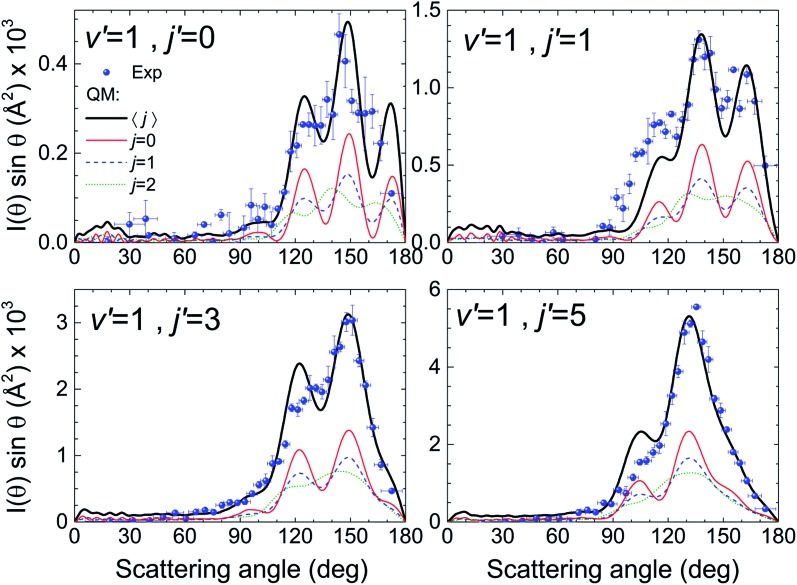
Comparison between the experimental angular distributions (blue solid circles) and their theoretically simulated counterparts (continuous black line) for four HD(*v*′, *j*′) rovibrational states at 1.97 eV mean collision energy. The simulation implies the averaging of the differential cross sections (multiplied by sin *θ*) over the experimental collision energy distribution and angular resolution. The simulation also implies consideration of the weighted contributions from the reaction with D_2_ in the rotational states populated under the experimental conditions. The corresponding contributions are indicated in each panel by the red line (*j* = 0), the blue line (*j* = 1) and the green line (*j* = 2) such that their sum yields the black line.

We will base our discussion on the state-to-state DCSs for the H + D_2_(*v* = 0, *j*) → D + HD(*v*′ = 1, *j*′) reactive encounters, where (*v*, *j*) and (*v*′, *j*′) represent the vibrational and rotational quantum numbers for D_2_ and HD, respectively. As the experiment has been carried out using *n*-D_2_ co-expanded through a nozzle, the resulting DCSs contain contributions from the three lowest rotational states populated in the D_2_ molecular beam (*j* = 0, 1 and 2). Fig. 1 illustrates the agreement between the experimental results and the QM calculations for the indicated HD rovibrational states. In all cases, to simulate the experimental DCSs, the QM results have been averaged over the experimental collision energy spread and, more importantly, over the D_2_ experimental rotational distribution (0.39, 0.31 and 0.29 for *j* = 0, 1 and 2, respectively). The respective contributions of each of the three rotational states are also shown in the figure, such that summing these contributions one obtains the simulated curve. It can be clearly seen that, in all cases, the shapes of the rotational contributions for *j* = 0 and 1 are quite similar, with the oscillations being more clearly defined for the initial *j* = 0. It is also worth noticing that the respective maxima for each of these two contributions, the most significant ones, coincide at the same angles. Had it been otherwise – the oscillations being out-of-phase with each other – the interferences would have been unobservable.

**Fig. 2:**
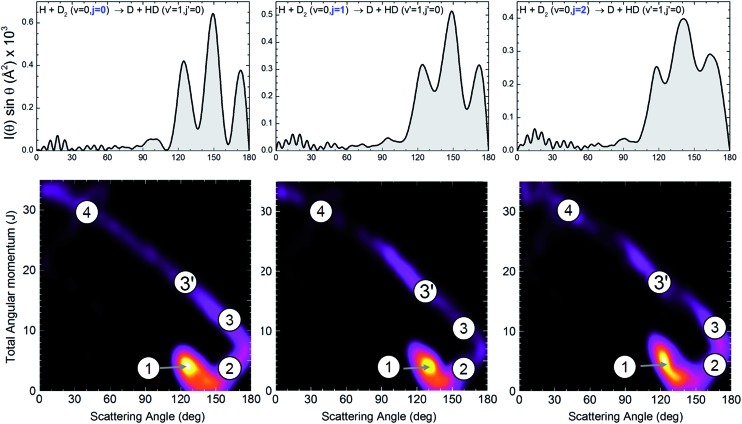
Angular distributions and deflection functions for the H + D_2_(*v* = 0, *j* = 0–2) → D + HD(*v*′ = 1, *j*′ = 0) reaction at 1.97 eV collision energy. The top panels show the QM angular distributions for the three rotational states populated in the experiment. The bottom panel shows the state-to-state quasiclassical deflection functions, *D*_r_(*J*, *θ*). Although the oscillation pattern is clearly affected by the initial rotational state, the QCT deflection functions are remarkably similar. The quasiclassical mechanisms are labelled in the figure as 1 (*ear*), 2, 3, 3′ and 4 (the last three form the *spiral*).^25,26^ The *spiral* mechanism displays a clear correlation between *J* and *θ* that extends over the whole range of scattering angles. The sketches of the mechanisms that correspond to the labelled regions of *D*_r_(*J*, *θ*) are displayed in Fig. S1, ESI.†

The dependence of the DCS with *j* is made more evident in the top panels of Fig. 2, where the DCSs are shown for HD(*v*′ = 1, *j*′ = 0) formation at 1.97 eV collision energy (without averaging over the collision energy spread). The angular distributions for *j* = 0, 1 and 2 bear a common resemblance, with very similar forward and sideways regions and oscillations in the backward region, with maxima at the nearly the same angles (*θ* ≈ 120, 150, and 175 degrees). However, the most salient feature is the progressive downgrading of the pattern with increasing rotational excitation. While the peak structure is very sharp for *j* = 0, the finger-like structure seems to smooth out for *j* = 1 and even more for *j* = 2. Consequently, the interference effect becomes weaker with rotational excitation.

The classical deflection function, that is, the joint reaction probability as a function of *J* and *θ*, defined as *D*_r_(*J*, *θ*) = (2*J* + 1)*P*_r_(*J*, *θ*)sin *θ*, where *P*_r_(*J*, *θ*) is the reaction probability into a solid angle element defined by *θ* at a total angular momentum, *J*, was proven to be an invaluable tool to analyse the interference pattern and to unravel the classical mechanisms behind the quantum interferences.^10^ Specifically, for initial *j* = 0, the interference pattern was observed whenever two separated groups of *J* gave rise to scattering at the same angles. We could therefore expect that the smoothed interference pattern would be associated with a significant change in its QCT deflection function. This is precisely what it is observed for higher *j*′ states. The gradual smoothing of the oscillations (Fig. 1) with increasing *j*′ can be traced back to differences in the quasiclassical *D*_r_(*J*, *θ*).^10^ Whereas for *j*′ = 0 there is a neat separation between the main two mechanisms, they merge at high *j*′,^10^ causing the interferences to vanish. Following the double-slit analogy, the two slits merge where *j*′ is sufficiently high.

However, the calculated *D*_r_(*J*, *θ*) for *j* = 1 and 2, shown in the bottom panels of Fig. 2, appear to be almost identical to that of *j* = 0, with small differences. Regardless of the initial state, the “*ear*” (labelled as 1), the mechanism 2 and the “*spiral*” mechanisms^25,26^ (labelled as 3, 3′ and 4) coexist; and, accordingly, it could be expected that they behave similarly as far as the interferences between different mechanisms are concerned. The *ear* mechanism corresponds to trajectories with small impact parameters, attacking angles far from collinearity and a T-shape transition state. The *spiral* mechanism, in turn, shows a strong *J*–*θ* correlation and is associated with nearly collinear transition states (see Fig. S1, ESI†). For the initial and final states displayed in Fig. 2, the magnitude of scattering is dominated by the *ear* mechanism with smaller contributions from the *spiral* one, although with a strong imprint *via* interferences between different types of trajectories, ultimately responsible for the finger-like structures that appear in the QM and experimental DCSs.^10^ It is evident that the classical deflection functions, as are depicted in the bottom panels of Fig. 2, cannot explain the difference in the amplitude of the oscillation pattern that therefore must be due to a different quantum effect.

As shown in the Methods section, the QM DCS expression comprises two kinds of summation: one coherent over the different partial waves contributing to scattering, and a second, incoherent, over the reagent D_2_ and product HD helicity quantum states, Ω and Ω′. The complete characterization of the asymptotic states of a closed-shell diatomic molecule, such as the D_2_ molecule, requires the helicity. Internal states with different values of Ω are asymptotically degenerate and cannot be isolated in typical scattering experiments. On the other hand, the different Ω states are associated with different distributions of the internuclear axis. For *j* ≠ 0, small values of |Ω| imply head-on collisions, whereas values of |Ω| close to *j* involve side-on approaches. Therefore, it can be expected that various Ω states will not necessarily behave alike during the collision process. Thus, they give rise to stereodynamical preferences.

The number of Ω states for a given *j* is 2*j* + 1, which implies that, for *j* = 0, the only possible projection of the total angular momentum onto the approach direction (in the body-fixed frame) is Ω = 0 and, hence, *j* = 0 corresponds to a pure state. For *j* = 1 and 2 there are three and five Ω states, respectively. In the absence of a field that breaks the degeneracy and of any specific preparation of the reactants, the asymptotic rovibrational states for *j* ≠ 0 reactant molecules are given by an incoherent mixture of the possible |*v*, *j*, Ω states – which are associated with different internuclear axis distributions – where each element carries the same weight.

**Fig. 3:**
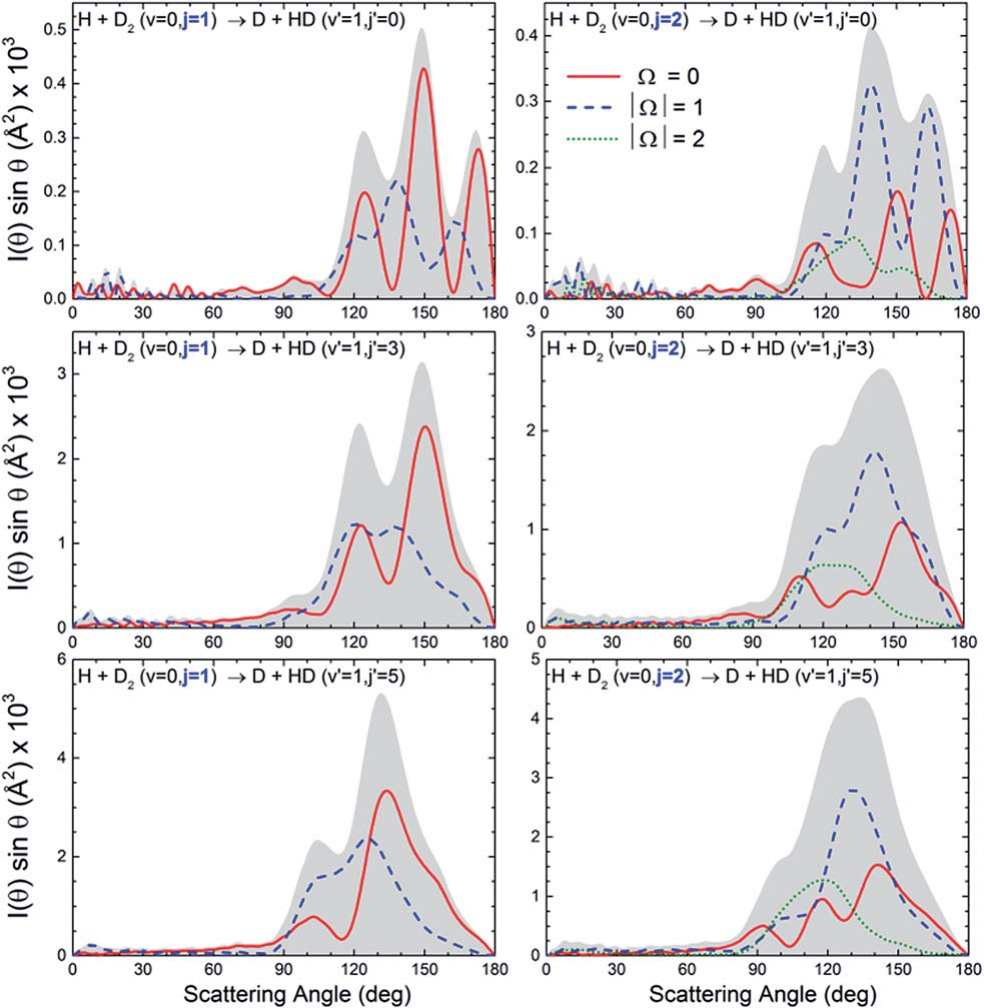
QM DCS(|Ω|) for the H + D_2_(*v* = 0, *j* = 1 and 2) → D + HD(*v*′ = 1, *j*′) reactions. The interference pattern, partially blurred as *j* increases, clearly reappears when the contributions due to the different |*v*, *j*, Ω states are separated.

At this point, it seems pertinent to examine the DCS, and particularly the backward structures, attributable to a single reactant's states, |*v*, *j*, Ω. Addressing this question implies the consideration of the DCS resolved in |Ω| values. Since DCS(Ω) = DCS(–Ω), we need to consider only the possible absolute values of the helicity. Such functions, which will be indicated as DCS(|Ω|), are presented in Fig. 3 for selected state-to-state processes. As can be seen, the position of the peaks and the overall shape of the DCS(|Ω|) depend strongly on Ω. For all the final (*v*′, *j*′) states examined, the finger-like pattern is sharper for Ω = 0, less so for |Ω| = 1, and almost absent for |Ω| = 2. But more importantly, as the global DCS is the incoherent sum of the DCS(|Ω|), averaging over Ω washes out the oscillation pattern to a considerable extent, and this is the main reason for the apparent progressive downgrading of the interference pattern with increasing *j*. An additional reason is the vanishing of the oscillation patterns for larger values of Ω.

**Fig. 4:**
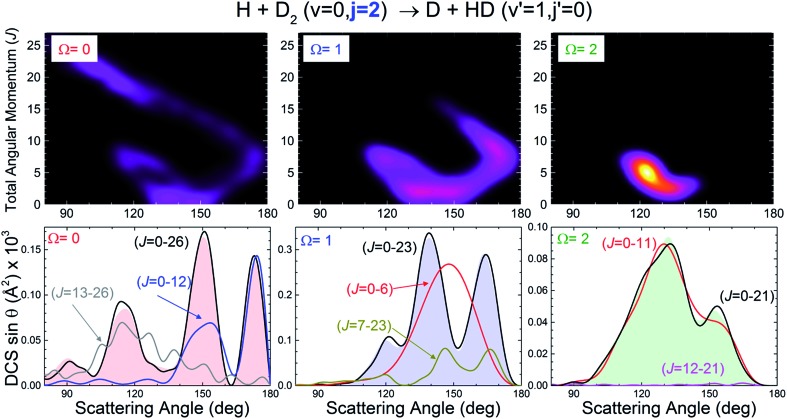
Origin of multiple peaks in backward scattering of HD(*v*′ = 1, *j*′ = 0, Ω) products for initial *j* = 2. The top panels show the joint QCT *J*–*θ* deflection function resolved in Ω, (2*J* + 1) *P*_r_(*J*, *θ*; Ω) sin *θ*, analogous to that displayed in panel (b) of Fig. 1. The bottom panels show the decomposition of the QM angular distributions from the contributions of various sets of *J*. The notation DCS(*J*_1_ – *J*_2_) means that the DCS is constructed by including partial waves in the range [*J*_1_, *J*_2_] including the respective cross terms. The global DCS(|Ω|) is depicted as a shaded background.

More information about the underlying mechanisms causing the observed behaviour for different Ω can be obtained from the inspection of the respective quasiclassical deflection function resolved in Ω, *D*_r_(*J*, *θ*; |Ω|). The results for *j* = 2 are shown in the top panels of Fig. 4. For Ω = 0 both the *spiral* and *ear* mechanisms coexist and have a similar importance. As will be explained below, this causes interferences that lead to three peaks of similar height. For |Ω| = 1, the sideways scattering part of the *spiral* has essentially vanished and the peak closer to the sideways region shrinks and survives as a shoulder. For |Ω| = 2, only the *ear* mechanism remains and, therefore, there are no interferences and the angular distribution is essentially characterized by a single peak at around 120 degrees. Similar results are obtained when the *D*_r_(*J*, *θ*; Ω) are calculated for *j* = 1 (see Fig. S2, ESI†). Note that the piecewise decomposition of the deflection function in various Ω allows us to unravel the preferred mechanism for the associated internuclear axis distributions. Summation over Ω, which gives rise to an isotropic axis distribution, leads to deflection functions remarkably similar to that found in the *j* = 0 case, as shown in the bottom panels of Fig. 2.

The breakdown of the DCS(|Ω|) in contributions from different partial waves is shown in the lower panels of Fig. 4. For Ω = 0 the situation is similar to that observed for *j* = 0. The most backward peak results from the interference of mechanisms (2) and (3). The peak appearing at 150 degrees is caused by the interference of mechanisms (1) and (3′). Finally, the third peak at 115 degrees, is an outcome of the interference of mechanisms (1) (with *J* ≥ 5) and (4). For |Ω| = 1, only two clear peaks remain. The outmost backward peak has the same origin as in the case of Ω = 0, while the second one comes from the interference of the lowest *J* values of mechanism (1) with some residual scattering in (3′). The stump at 120 degrees has the same origin as that observed at 115 degrees for Ω = 0. Finally, for |Ω| = 2, practically all scattering is caused by the *ear* mechanism, although the shoulder at 155 degrees is the result of the interference between two very weak sources which would be the remnants of mechanisms (2) and (3). The fact that the shape of the DCS(|Ω|) and, in particular, that the amplitude of the interferences depends strongly on Ω means that the amplitude of those interferences can be potentially controlled in experiments such as those suggested in ref. 27. The presence of interferences cannot modify the total reactive flux (area of the DCS). Hence, the presence of a sharp peak that stems from interferences between two mechanisms should also give rise to troughs, such as that appearing at 150 degrees for |Ω| = 1 where all relevant partial waves are included (*J* = 0–23).

**Fig. 5:**
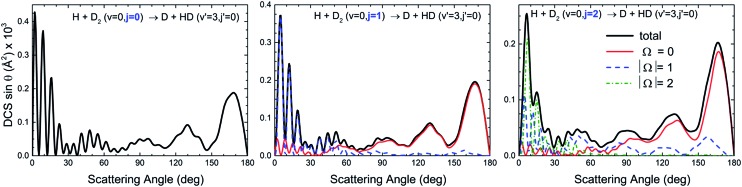
QM DCS(|Ω|) and angular distributions and deflection functions for the H + D_2_(*j* = 0–2) → D + HD(*v*′ = 3, *j*′ = 0) reaction.

In contrast, we examine the behaviour displayed for HD products in a *v*′ = 3 state, where no oscillations were experimentally observed and no interferences could be found in the angular distribution for *j* = 0. For the initial D_2_(*v* = 0, *j* = 0) state, no interferences were observed in the backward region.^10^ It serves as a counter-example because it represents a typical situation where the different features of the angular distributions can be attributed to the contribution of different groups of partial waves without interferences between them. In this case, the peaks observed in the sideways and backward directions could be reproduced by QCT calculations and attributed to groups of partial waves. Fig. 5 demonstrates that the rotational excitation of D_2_ barely has any effect on the shape of the angular distribution. This behaviour is not surprising because the scattering in the *v*′ = 3 manifold is only due to the *spiral* mechanism and, therefore, the evolution of the DCS(|Ω|) functions agrees point by point with the features of this mechanism: collinear approaches and a progressive tilting of the internuclear axis as the scattering moves from the backward to the forward directions. Accordingly, backward scattering mainly correlates with Ω = 0 collisions and the preeminent character of the DCS(Ω = 0) contribution is transferred to DCS(|Ω|) functions corresponding to increasingly larger values of the helicity as scattering moves into the forward hemisphere.

## Conclusions

Whenever different classical scattering mechanisms coexist, each of them characterized by different sets of total angular momentum and leading to similar scattering angles, they will interfere with each other giving rise to a characteristic oscillation pattern in the angular distribution, similar to what is found in the multiple-slit experiment. We have shown that the rotational excitation of the reactants has a strong influence on the sharpness of the oscillatory patterns. However, the ultimate reason is not the progressive disappearance of interferences with increasing *j*, but rather the incoherent sum of contributions from different helicity states of the reagents, associated with different internuclear axis distributions.

The oscillations become much more evident when the DCSs from different helicity states are analysed separately. In particular, we have found that the shape of the angular distributions depends on Ω, causing the oscillation pattern to be most prominent for small values of Ω (head-on collisions) where the *spiral* and the *ear* mechanisms coexist. For large values of Ω (side-on encounters) only the *ear* mechanism survives and thus oscillations are no longer observed.

In turn, the appearance or disappearance of certain mechanisms for selected values of the helicity stems from their different stereodynamical requirements. As only those mechanisms that correlate with the same value of Ω can interfere, we can conclude that it is the stereodynamics that lies behind the structure of the DCS(Ω) functions or, in other words, the stereodynamics determines the extent and nature of the interferences between mechanisms when *j* ≠ 0.

An important conclusion of this work is that, whereas the analysis of the interferences with rotationless reactants serves to reveal the existence of competing mechanisms, their analysis and decomposition in helicities in the case of *j* ≠ 0 makes it possible to determine the stereodynamical preferences of each of those mechanisms, that is, their preferences for certain approach directions.

We have also examined the behaviour of collisions leading to HD(*v*′ = 3, *j*′) where only one mechanism is observed. An increase in the rotational energy of the reactants has no effect on the shape of the DCS. Moreover, the different values of Ω tend to correlate with scattering into different regions of the angular distribution, according to the stereodynamical requirements of the single mechanism that is operative.

The calculations presented here are for the H + D_2_(*v* = 0, *j* = 0, 1 and 2) → HD(*v*′, *j*′) + D reaction for which we have experimental measurements of the differential cross sections that can be compared to fully quantum calculations using a highly accurate PES. This comparison gives us confidence in the conclusions we have stated above. However, the same behaviour is expected for any elementary chemical reaction in which we have more than one scattering mechanism leading to state-resolved products being scattered into the same solid angle. In this sense, the PES acts as an intrinsic molecular interferometer. According to our calculations, even in the absence of QM state-to-state results for more complex systems, quasiclassical deflection functions could be used to discern whether the QCT DCS might be reliable or if interference phenomena are expected to modify its shape.
