# Correction: Effects of reagent rotation on interferences in the product angular distributions of chemical reactions

**DOI:** 10.1039/c6sc90050j

**Published:** 2016-07-21

**Authors:** P. G. Jambrina, J. Aldegunde, F. J. Aoiz, M. Sneha, R. N. Zare

**Affiliations:** a Departamento de Química Física I , Facultad de Química , Universidad Complutense de Madrid , 28040 , Spain . Email: aoiz@quim.ucm.es; b Departamento de Química Física , Universidad de Salamanca , Salamanca , Spain; c Department of Chemistry , Stanford University , Stanford , California 94305-5080 , USA . Email: zare@stanford.edu

## Abstract

Correction for ‘Effects of reagent rotation on interferences in the product angular distributions of chemical reactions’ by P. G. Jambrina *et al.*, *Chem. Sci.*, 2016, **7**, 642–649.



## 

In the Acknowledgements section the grant number CHE-1464646 should have been CHE-1464640.

The Royal Society of Chemistry apologises for these errors and any consequent inconvenience to authors and readers.

