# Supplementary material for: Effects of reagent rotation on interferences in the product angular distributions of chemical reactions
Source: Chem Sci. 2015 Oct 5;7(1):642–9. doi: 10.1039/c5sc03373j (PMC5523120; doi:10.1039/c5sc03373j)
Supplement: Supplementary file 1 [file SC-007-C5SC03373J-s001.pdf]

Electronic Supplementary Material (ESI) for Chemical Science.
This journal is © The Royal Society of Chemistry 2015

Effects of reagent rotation on interferences in the product angular distributions of chemical reactions

P. G. Jambrina,[a] J. Aldegunde[b], F. J. Aoiz*[a], M. Sneha[c], R. N. Zare*[c]

[a]Departamento de Química Física I, Facultad de Química, Universidad Complutense de Madrid, 28040, Spain. e-mail: aoiz@quim.ucm.es
[b]Departamento de Química Física. Universidad de Salamanca. Salamanca. Spain.
[c]Department of Chemistry, Stanford University, Stanford, California 94305-5080, USA. e-mail: zare@stanford.edu

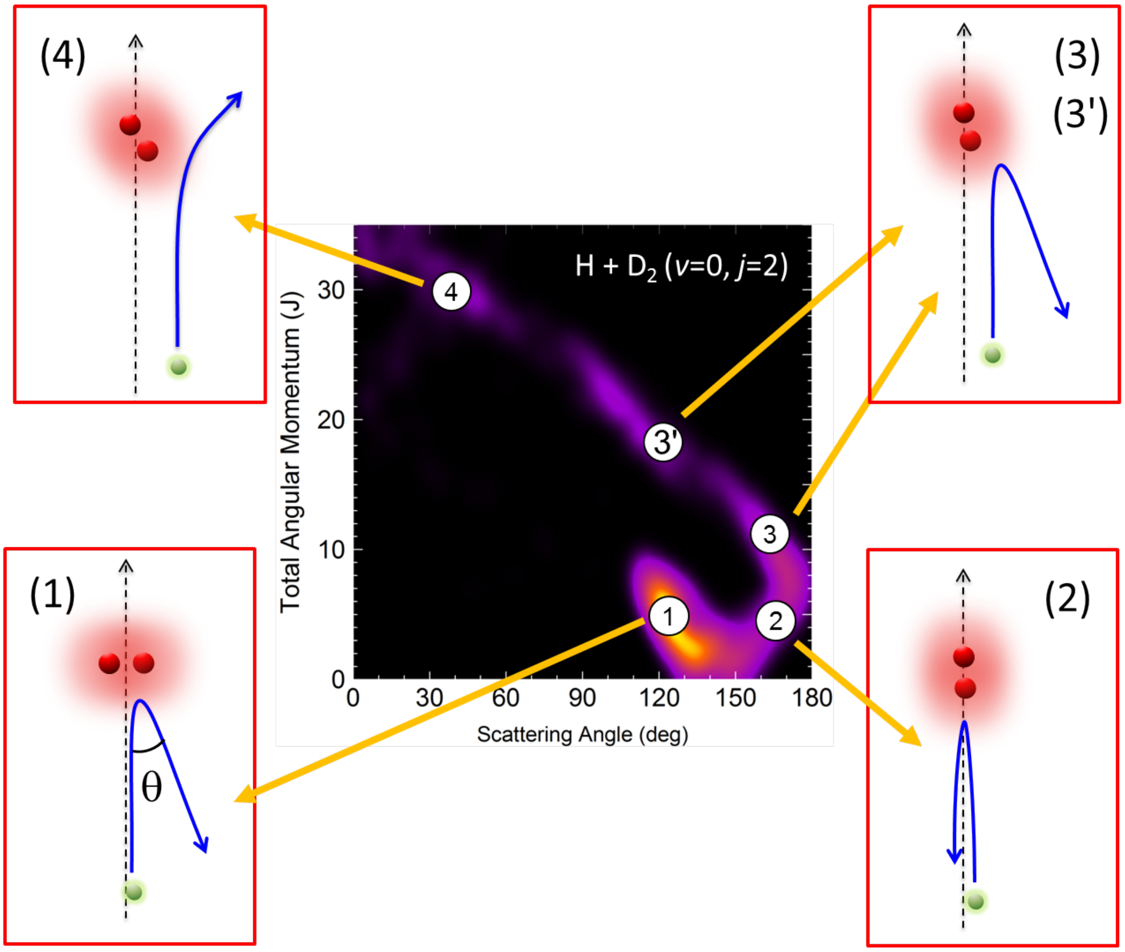

Figure S1: QCT J-θ deflection function resolved in Ω, (2J + 1) P_r (J, θ; Ω) sin θ for the H + D_2 (v=0, j=2) → D + HD (v=1, j=0) reaction at E_{coll} = 1.97 eV. Sketches of the most characteristic quasiclassical mechanisms are labelled in the figure as 1 (ear), 2, 3, 3' and 4 (the last three form the spiral). [1, 2] Sketches displaying these mechanisms are also displayed. The mechanism labelled as (3) and (3') are similar; the former with slightly smaller impact parameters and attacking angles somewhat closer to linearity.

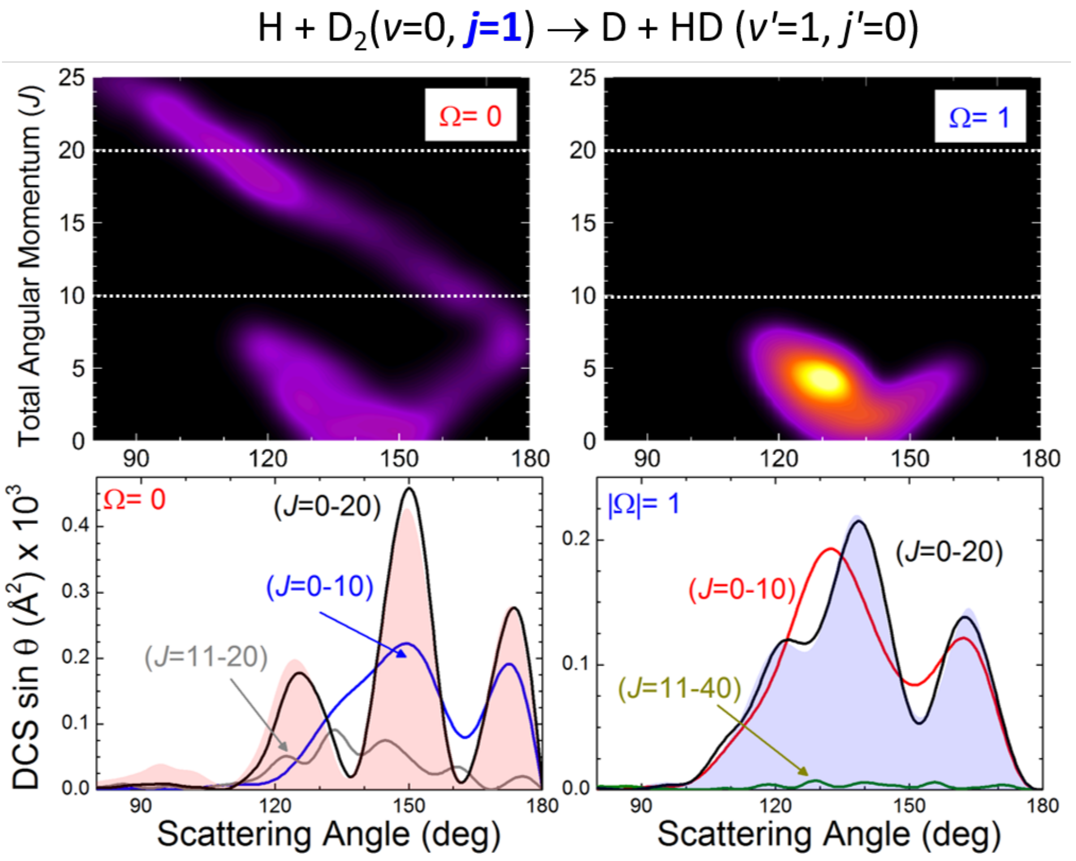

Figure S2: Origin of the multiple peaks in backward scattering for j=1. The top panels show the joint QCT J-θ deflection function resolved in Ω, $(2J + 1)\, P_r\,(J, \theta; \Omega)\, \sin\theta$. The bottom panels show the decomposition of the QM angular distributions from the contributions of various sets of J. The notation DCS(J_1–J_2) means that the DCS is constructed by including partial waves in the range $[J_1, J_2]$ and the corresponding cross terms. In each case, the shaded curve corresponds to the global DCS $|v, j, \Omega\rangle$ state.

References:

1. S. J. Greaves, D. Murdock and E. Wrede, *J. Chem. Phys.*, 2008, **128**, 164307.
2. S. J. Greaves, D. Murdock, E. Wrede and S. C. Althorpe, *J. Chem. Phys.*, 2008, **128**, 164306.
